# Superior Cerebellar Arteries Originating from the Posterior Cerebral Arteries but Normal Course of the Oculomotor Nerves

**DOI:** 10.7759/cureus.2932

**Published:** 2018-07-05

**Authors:** Dominic Dalip, Joe Iwanaga, Marios Loukas, Rod J Oskouian, R. Shane Tubbs

**Affiliations:** 1 Seattle Science Foundation, Seattle, USA; 2 Medical Education and Simulation, Seattle Science Foundation, Seattle, USA; 3 Anatomical Sciences, St. George's University, St. George's, GRD; 4 Neurosurgery, Swedish Neuroscience Institute, Seattle, USA; 5 Neurosurgery, Seattle Science Foundation, Seattle, USA

**Keywords:** posterior cerebral artery, superior cerebellar artery, basilar artery, variations, anatomy

## Abstract

The posterior cerebral artery (PCA) is a branch of the terminal part of the basilar artery and perfuses the temporal lobes, midbrain, thalamus, and the posterior inferior portion of the parietal lobes. It is divided into P1-P4 segments. Variations in the P1 segment of the PCA are important to neurosurgeons when performing surgery, for example, on basilar tip aneurysms. We report bilateral superior cerebellar artery (SCA) arising from the P1 segment of the PCA. Such a configuration appears to be uncommon but should be kept in mind by neurosurgeons, neurointerventionalists, and neuroradiologists.

## Introduction

The temporal lobes, midbrain, thalamus, and the posterior inferior portion of the parietal lobes are supplied by the posterior cerebral artery (PCA) which is a branch of the terminal part of the basilar artery [1]. The superior cerebellar artery (SCA) usually originates from the basilar artery [2]. The superior vermis, the tectum, and superior surface of the cerebellar hemispheres are supplied by the SCA. Variations in the P1 segment of the PCA are important to neurosurgeons, neurologists and neuroradiologists alike [2,3]. Herein, we report an uncommon case of the left and right SCA arising from the P1 segments of the PCA in a cadaver.

## Case presentation

**Figure 1:**
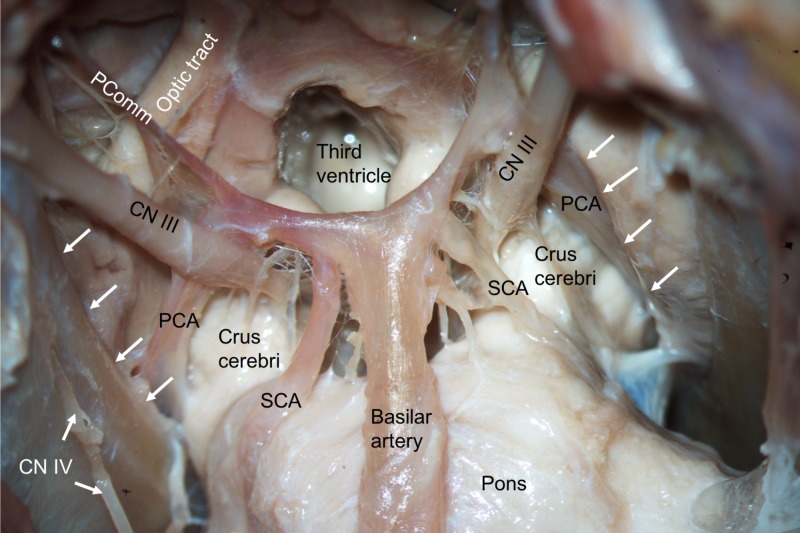
Inferior view of the basal brain and associated neurovascular structures. Note the posterior cerebral arteries (PCA), superior cerebellar arteries (SCA), oculomotor (CN III) and trochlear nerves (CN IV) and posterior communicating artery (PComm). Note that the left and right SCAs arise from the P1 segments of the PCA.

During routine dissection of the skull base of a male cadaver, an unusual origin of the left and right SCA was observed. The cadaver was 89-years-old at death. Following removal of the bony parts of the skull base, the dura mater was opened. From an inferior view (Figure 1) it was noted that the left and right SCA originated from the P1 segments of the left and right PCA. In normal fashion, the oculomotor nerve passed between the PCA and SCA on both sides (Figure 1). The SCA on both sides left the PCA and traveled over the crus cerebri at their junction with the pons to extend to the cerebellum, under the free edge of the tentorium cerebelli, in a typical fashion. No other intracranial anatomical variations or intracranial pathology was observed.

## Discussion

Our case demonstrates an unusual origin of the left and right SCA from the P1 segments of the PCA. The PCA is a branch of the terminal part of the basilar artery that perfuses the temporal lobes, midbrain, thalamus, and the posterior inferior portion of the parietal lobes [1]. The PCA is divided into four segments (P1, P2, P3, and P4) and branches into the posterior communicating artery, choroidal branches from P2 segment, perforating branches, and temporal lobe branches [1]. The P1 segment is the part of the PCA that extends from the basilar artery to the posterior communicating artery. The oculomotor nerve usually passes between the P1 segment of the PCA and the SCA. The portion of PCA from the posterior communicating artery to the posterior edge of the midbrain is termed the P2 segment. The P3 segment continues from the P2 segment to the calcarine fissure. The anterior calcarine sulcus marks the beginning of the P4 segment and continues to the cortical surface where this segment terminates [3,4]. Caruso et al. [3] reported that the variations in the P1 segment are rare and occur in only 3% of cases [3]. One case of P1 segment duplication was established in this study and two other cases were described in the literature [3, 5-6]. There were two left-sided P1 segments of the PCA. The P1 segment originated as a single vessel, then separated 4 mm after its branching from the basilar artery. The P1 segment of the PCA and the SCA formed a common trunk bilaterally in a single case [3].

The SCA can be divided into three main portions: interpeduncular-crural, ambient and quadrigeminal. The first portion of the SCA is the interpeduncular-crural portion, which angles laterally from the basilar artery to meet the anterolateral brain stem. At this point, the artery is inferior to the oculomotor nerve [7]. The ambient portion is the second part of the SCA that passes posteriorly into the ambient cisterns [2]. The marginal branch arises from this portion and is the first large branch of the SCA. Distally, two or three small hemispheric branches arise. The final portion of the SCA is the quadrigeminal part and this artery forms an anastomosis with the superior cerebellar arteries to perfuse the tectum. This portion gives rise to the terminal branch of the SCA to the superior vermis. There are a variety of variations of the SCA. Twenty-eight out of 100 patients displayed duplication of the SCA with 8% being bilateral. Furthermore, unilateral triplication was reported in two patients [2]. Hardy et al. [7] described seven cases where the SCA arose from a duplicated trunk. In 20% of cases, the SCA was found to be duplicated [7,8]. Absence of the SCA is rare, but can be observed [9]. Hardy et al. [7] found 2/50 (4%) SCAs arising from the proximal PCA. However, in both cases, the arteries crossed over the oculomotor nerve. In our case (Figure 1), the oculomotor nerve passed between the PCA and SCA in typical fashion [10].

## Conclusions

The SCA was found to branch from the P1 segment of the PCA during dissection of a cadaver. Such a configuration appears to be uncommon but should be kept in mind by neurosurgeons, neurointerventionalists, and neuroradiologists.
